# Effects of Biochar Combined with Nitrogen Fertilizer Reduction on Rapeseed Yield and Soil Aggregate Stability in Upland of Purple Soils

**DOI:** 10.3390/ijerph17010279

**Published:** 2019-12-31

**Authors:** Xiaoqin Tian, Zhuo Li, Longchang Wang, Yifan Wang, Biao Li, Meichun Duan, Bangyan Liu

**Affiliations:** 1 College of Agronomy and Biotechnology, Southwest University, Chongqing 400715, China; a865257025@163.com (X.T.); w951352241@email.swu.edu.cn (Y.W.); libiao08.05@163.com (B.L.); duanmc@swu.edu.cn (M.D.); bangyan_liu@126.com (B.L.); 2 Key Laboratory of Eco-environments in Three Gorges Reservoir Region, Ministry of Education, Chongqing 400715, China; 3 Engineering Research Center of South Upland Agriculture, Ministry of Education, Chongqing 400715, China; 4 Crop Research Institute, Sichuan Academy of Agriculture Sciences, Chengdu 610066, China; lizhuo_2000@sina.com

**Keywords:** upland purple soil, biochar, rapeseed yield, soil aggregate, nitrogen fertilizer

## Abstract

Reduction of soil fertility and production efficiency resulting from excessive application of chemical fertilizers is universal in rapeseed-growing fields. The main objective of our study was to assess the effects of biochar combined with nitrogen fertilizer reduction on soil aggregate stability and rapeseed yield and to identify the relationship between yield and soil aggregate stability. A two-factor field experiment (2017–2019) was conducted with biochar (0 (C_0_), 10 (C_10_), 20 (C_20_) and 40 t·ha^−1^ (C_40_)) and nitrogen fertilizer (180 (N_100_), 144 (N_80_) and 108 kg N·ha^−1^ (N_60_)). Experimental results indicated that under N_100_ and N_80_ treatments, C_10_ significantly increased the macro-aggregates (R_0.25_), mean weight diameter (MWD) and geometric mean diameter (GMD) of soil water stable aggregate by 14.28%–15.85%, 14.88%–17.08% and 36.26%–42.22%, respectively, compared with C_0_. Besides, the overall difference of the soil water-stable aggregate content in 2–5 mm size range among nitrogen treatments was significant under the application of C_10_, which increased by 17.04%–33.04% compared with C_0_. Total organic carbon (TOC) in R_0.25_ of soil mechanical-stable aggregates was basically all increased after biochar application, especially in 0.25–1 mm and 1–2 mm aggregates, and had an increasing trend with biochar increase. C_10_ significantly increased rapeseed yield by 22.08%–45.65% in 2019, compared with C_0_. However, the reduction of nitrogen fertilizer reduced the two-year average rapeseed yield, which decreased by 11.67%–31.67% compared with N_100_. The highest yield of rapeseed was obtained by N_100_C_10_ in two consecutive years, which had no statistical difference with N_80_C_10_. However, the two-year yields of N_80_C_10_ were all higher than those of N_100_C_0_ with increase rate of 16.11%, and which would reduce 35.43% nitrogen fertilizer in the case of small yield difference, compared with the highest yield (2.67 t·ha^−1^) calculated by multi-dimensional nonlinear regression models. The regression analysis indicated R_0.25_, MWD and GMD had the strong positive associations with rapeseed yield, whereas percentage of aggregate destruction (PAD_0.25_) had a significant negative correlation with rapeseed yield. This study suggests that the application of biochar into upland purple soil could improve soil structure, increase the content of TOC in macro-aggregates under nitrogen fertilizer reduction as well as replace part of nitrogen fertilizer to achieve relatively high rapeseed yield.

## 1. Introduction

Soil aggregates are the material basis of soil fertility by reducing soil erosion, adjusting air permeability, water infiltration and nutrient cycling [1,2] and contributing to soil functions. It regulates soil physical, chemical and biological processes, and thus affects the function of soil organic matter and fertility [3]. At the same time, it is also largely responsible for soil structural stability, which is fundamental for improving crop yield, preventing soil degradation and reducing environmental pollution [4]. The quantity, distribution and spatial arrangement of soil aggregates of various sizes have an important role in controlling soil pore distribution, determining soil hydraulic properties and permeability and influencing soil microbial activity as well as nutrients maintenance and supply [5]. However, soil aggregation is controlled by many factors, such as soil organic carbon (SOC), soil animals, microorganisms and plant root systems. The soil aggregates and SOC are interdependent and closely linked and interrelated. Soil organic matter is one of the major constituents in the formation of aggregates by serving as a major binding agent in the formation and stabilization of aggregates, while on the other hand soil aggregates protect soil organic matter (SOM) from mineralization because they are less vulnerable to microbial, enzymatic and physical degradation [6]. As one of the main producing areas of rapeseed in China, the annual rapeseed planting area and output in southwest regions account for 20%–30% of the country. Since the 1970s, some unreasonable agricultural management measures with excessive use of chemical fertilizers and imbalance of input ratio of nitrogen, phosphorus and potassium resulted in the depletion of soil organic carbon, breakdown of soil structure and decline of soil productivity in uplands in southwest China [7]. In 2010, China has become the world’s largest chemical fertilizer consumption country. Nitrogen fertilizer accounts for about 60% of the chemical fertilizer and the annual nitrogen fertilizer consumption accounts for more than 35% of the world’s total consumption, and it is increasing year by year. Therefore, it is very urgent to explore economic and effective fertilization measures for improving soil structure, soil fertility and crop productivity in upland purple soil in southwest China.

Crop straw is a kind of renewable bio-resource. It is rich in organic carbon, nitrogen, phosphorus, potassium and other mineral nutrients. However, the discarding and burning of straw will cause environmental pollution and recourse waste. Recently, biochar, a significant carbonaceous component arising from the incomplete combustion of various organic precursors such as crop straw, has attracted increasing attention in China as its role in achieving the sustainable development of resources, environment and agriculture. Various studies have shown the beneficial effects of biochar as a soil amendment to reduce greenhouse gas emission and improve heavy metal pollution [8,9]. In addition, biochar can enhance soil microorganism activity and indirectly change the mineralization rate of SOC, which in turn influences the formation process of soil aggregates [6]. For example, Joseph et al. [10] reported that biochar significantly improved water-stable aggregate stability in albic soils and the biochar application had positive effects on mean weight diameter (MWD) of water-stable aggregates, SOC and total nitrogen content (TN) within aggregates. Hence, biochar has been used not only as an amendment to maximize the efficient use of straw nutrients, but also as an important technical means to improve the soil environment, increase crop yield and quality, and achieve fertilizer reduction and efficiency increase. However, no consistent conclusions currently exist on the effects of biochar on the amount and stability of soil aggregates in previous studies in response to changing biochar species, biochar dosage or soil conditions. Joseph et al. [10] found an increase in aggregate formation when biochar was applied to albic soils. Contrariwise, Zhou et al. [11] did not observe any improvement in soil aggregate in sandy loam soil after sole biochar application. Moreover, Heikkinen et al. [12] reported that properties of the biochar depended on feedstock type, with significantly improved aggregate stability and reduced colloid detachment with hydrothermal carbonization biochars, but no effect with slow pyrolysis biochars. These differences found in soil aggregate after biochar application may be related to many complex interactions and bonding mechanisms of biochar, clay minerals and native SOM [13]. In China, research on effect of biochar on soil aggregates have been carried out on a wide range of soil types, such as ferrallitic soil [14], leached soil [15] and semi-hydromorphic soil [16,17]. However, for upland lithomorphic soil (purple soils), the effect of biochar based on nitrogen fertilizer reduction on soil aggregate stability is not well known. Besides, based on the short-term conditions, whether the biochar can replace nitrogen fertilizer still lacks sufficient practice verification.

Therefore, the aim of this study was to (1) assess the impacts of biochar combined with nitrogen reduction on soil mechanical and water-stable aggregate distribution, soil aggregate stability and total organic carbon (TOC) content in different sizes of mechanical aggregates in upland of purple soils; and (2) determine whether the application of biochar can replace 20%-40% nitrogen fertilizers to increase rapeseed production in upland of purple soils in southwestern China. Experimental results are expected to provide important theoretical basis and technical support for the rational use of biochar in rapeseed production in upland of purple soils in southwestern China, improving soil quality and achieving high yield and high efficiency of rapeseed.

## 2. Materials and Methods

### 2.1. Study Site

The two-years field experiments were conducted during the two successive rapeseed seasons of 2017–2018 and 2018–2019 in Jiangnan village, Yunyang country, Chongqing, China (108°54′ E, 30°55′ N, elevation 700 m). The experimental field was located in a subtropical monsoon humid climate zone. The mean annual temperature and rainfall were 18.4 °C and 1100.1 mm, respectively, with rainfall mainly occurring during June to August. The test soil is purple soil, and its basic properties were as follows: pH 7.29 (soil:water of 1:5), total N 0.94 g·kg^−1^, total C 7.14 g·kg^−1^, available N 37.45 mg·kg^−1^, available P 2.36 mg·kg^−1^ and available K 72.58 mg·kg^−1^, respectively. 

### 2.2. Biochar Amended

A commercially produced biochar (purchased from the Nanjing Qinfeng Straw Technology Co., Ltd. (Nanjing, China)), which was made by pyrolysis of the rice (*Oryza sativa* L.) straw with limited oxygen supply at 500 °C for 2 h, was used. Its properties were as follows: total N 0.61 g·kg^−1^, total P 1.99 g·kg^−1^, total K 27.15 g·kg^−1^, total C 537.97 g·kg^−1^ and pH 8.70.

### 2.3. Field Experiment

A two-factor and multiple-level experiment was performed. The first factor was the application of biochar, including: 0 t·ha^−1^(C_0_), 10 t·ha^−1^ (C_10_), 20 t·ha^−1^ (C_20_) and 40 t·ha^−1^ (C_40_). Nitrogen fertilizer application was the second factor, including: 1) conventional application rate (180 kg N·ha^−1^; N_100_); 2) 80% of conventional application rate (144 kg N·ha^−1^; N_80_) and 3) 60% of conventional application rate (108 kg N·ha^−1^; N_60_). Winter rapeseed (Sanxiayou No. 5) was used in the experiment. Twelve treatments (4 × 3) were setup in a randomized block design with three replications. Each plot was 3 m × 6 m with a border (0.5 m wide) between plots. Following the local conventional fertilization, the fertilizer N of urea was applied according to different nitrogen fertilizer treatments during the rapeseed season, 40% of which was as a base fertilizer prior to seeding and the left 60% at the bolting stage. Both pure P and K for rapeseed were as a basal application of 90 kg P_2_O_5_ ha^−1^ and 90 kg K_2_O ha^−1^ through calcium superphosphate and potassium chloride, respectively, which were applied before ploughing and sowing. Biochar was added to the soil surface just at the beginning of the trial and thoroughly mixed with a depth of approximately 15 cm, and no further biochar was added for the duration of the study. Agricultural managements were same as those used on local farmlands. Rapeseed was planted on 21 October 2017 and on 16 October 2018, and was harvested on 1 May 2018 and on 1 May 2019.

### 2.4. Soil Sampling and Analysis 

Soil samples (0–20 cm) were collected at five points in each plot and then mixed (mass was about 1.5 kg) on 4 May 2019, and were sealed in plastic bags and transported to the laboratory as soon as possible. Soil samples were broken into small pieces to pass a 10 mm sieve and then air-dried, and used for soil aggregate analysis. Basic soil properties and biochar properties were determined using the methods suggested by Teng et al. [18]. Soil pH and biochar pH was determined by a glass electrode (solid-to-water ratio of 1:2.5), organic C in soil and biochar by dichromate oxidation with heating (K_2_Cr_2_O_7_–H_2_SO_4_), total N in biochar by the semi-micro Kjeldahl method, total P in biochar by a phosphomolybdic acid blue color method, total K in biochar by the NaOH melting-flame photometric method, alkali-hydrolyzable N in soil by the alkali solution diffuse method, available P in soil by the NaHCO_3_ (pH 8.5) extraction-phosphomolybdate blue spectro-photometric method and available K in soil by the NH_4_OAc extraction-flame photometer method. At maturing stage in 2018 and 2019, rapeseed in each plot was separately harvested to measure seed yield.

### 2.5. Aggregate Separation

A combination of wet and dry sieving methods was employed for soil aggregate classification [5]. The dry sieving method was used to determine soil mechanical-stable aggregate size distribution (ASD). To do so, 500 g air-dried soil samples were dry-sieved using a stainless-steel vibrating sieve set. Mechanical-stable aggregates of various sizes (>5, 2–5, 1–2, 0.25–1 and <0.25 mm) were obtained through dry sieving. Soil aggregates from each sieve were analyzed for organic carbon content. Wet sieving method was used to determine soil water-stable ASD. Briefly, 200 g air-dried soil was weighed and placed on the first sieve composed of 5, 2, 1 and 0.25 mm mesh sieves in a water bucket and was gently moistened for 5 min with distilled water at room temperature. The aggregates of various sizes were separated by moving the sieve vertical with an amplitude of 3 cm at a speed of 30 strokes·min^−1^ for 2 min after pre-wetting. After sieving, all aggregate-size fractions remaining on each sieve were collected and dried at 60 °C, and then weighed.

For the determination of ASD of soil mechanical-stable aggregates and water-stable aggregates, the weight ratio of aggregates of each sieve (>5, 2–5, 1–2, 0.25–1 and <0.25 mm) to the total weight of aggregates was calculated. Aggregate stability indices were denoted by >0.25 mm soil aggregates (R_0.25_), soil aggregate destruction (PAD_0.25_), mean weight diameter (MWD) and mean geometric diameter (GMD) [5].

(1) $${\mathrm{DR}}_{0.25}\;({\mathrm{WR}}_{0.25})\;=\;\frac{\sum_{i=1}^{n}\left(w_{i>0.25}\right)}{\sum_{i=1}^{n}\left(w_{i}\right)}\times 100\%,$$

(2) $$D\text{-}\mathrm{MWD}\;(W\text{-}\mathrm{MWD})\;=\;\sum_{i=1}^{n}\left(\bar{d_{i}}w_{i}\right),$$

(3) $$D\text{-}\mathrm{GMD}\;(W\text{-}\mathrm{GMD})\;=\;\exp\;\left[\frac{\sum_{i=1}^{n}m_{i}\ln\bar{d_{i}}}{\sum_{i=1}^{n}m_{i}}\right],$$

(4) $$\mathrm{PAD}0.25_{\;}=\;\frac{{\mathrm{DR}}_{0.25}-{\mathrm{WR}}_{0.25}}{{\mathrm{DR}}_{0.25}}\times 100\%,$$

where DR_0.25_ and WR_0.25_ are the proportion of >0.25 mm soil mechanical-stable aggregates and water-stable aggregates, respectively; D-MWD and W-MWD are the mean weight diameter of mechanical-stable aggregates and water-stable aggregates (mm), respectively; D-GMD and W-GMD are the mean geometric diameter of mechanical-stable aggregates and water-stable aggregates (mm), respectively; *m_i_* is mass in size fraction *i*; and *w_i_* is the proportion (%) of the total sample mass in size fraction *i* and $\bar{d_{i}}$ is mean diameter of size fraction *i*.

### 2.6. Statistical Analysis

Two-way analysis of variance (ANOVA) was performed by SPSS 17.0 (SPSS Inc., Chicago, IL, USA). Means were tested using the multiple-comparison performed by least significant difference (LSD) at *p* < 0.05. Excel 2018 (Office Software, Inc., Beijing, China) and SigmaPlot 12.5 (Systat Software, Inc., Erkrath, Germany) software were used for drawing figures.

## 3. Results

### 3.1. Effect of Biochar and Nitrogen Fertilizer on Soil Aggregate Content and Distribution

After a two-year field experiment, differences in the content and distribution of soil aggregates were observed among the different biochar and nitrogen fertilizer treatments (Table 1 and Table 2). As shown in Table 2, The particle size distribution of soil mechanical-stable aggregates for each nitrogen treatment was basically the same, mostly concentrated within the size ranges of >5 mm and 2–5 mm, and the content of soil mechanical-stable aggregates in size of 1–2 mm was the least. For each nitrogen treatment, biochar increased the soil mechanical-stable aggregate content in 1–2 mm size range, especially at the optimal dosage of 10 t·ha^−1^, and N_60_C_10_ significantly increased it by 46.00% compared to N_60_C_0_ (*p* < 0.05). This is mainly due to the reduction of percentages of soil mechanical-stable aggregates in size of <0.25 mm, with a decrease of 28.93%–32.58%. These results showed that the application of 10 t·ha^−1^ biochar for each nitrogen treatment could contribute to soil mechanical-stable aggregates with a change from <0.25 mm to 1–2 mm. 

It can also be seen from Table 2 that biochar increased the soil water-stable aggregate content in >5 mm size range for each nitrogen treatment, and the difference between N_80_C_40_ and N_80_C_0_, N_60_C_20_ and N_60_C_0_, N_60_C_40_ and N_60_C_0_ was significant (*p* < 0.05), with an increase of 24.43%–35.80%. In addition, the content of soil water-stable aggregates of 2–5 mm generally increased firstly and then decreased with the increase of biochar, but decreased slightly with the decrease of nitrogen fertilizer. The overall difference of the soil water-stable aggregate content in 2–5 mm size range for each nitrogen treatment was significant (*p* < 0.05) at the application of 10 t·ha^−1^ biochar, with the increase of 17.04%–33.04% compared with no biochar. This is also mainly due to the reduction of percentages of soil water-stable aggregates in size of <0.25 mm, with a decrease of 14.49%–30.88%. This indicated that biochar could effectively increase the content of soil water-stable aggregates in >5 mm and 2–5 mm size ranges for each nitrogen treatment, especially at the optimal dosage of 10 t·ha^−1^, and it could significantly promote the formation of soil water-stable aggregates of 2–5 mm size range from <0.25 mm size range.

### 3.2. Effect of Biochar and Nitrogen Fertilizer on Soil Stability Index

Except for PAD_0.25_, for each nitrogen fertilizer treatment, the effects of biochar on other soil stability index (DR_0.25_, WR_0.25_, D-MWD, D-GMD, W-MWD and W-GMD) reached a highly significant level (*p* < 0.01; Table 1). After the application of biochar, the difference in DR_0.25_ was not significant for the each nitrogen fertilizer treatment (*p* > 0.05), but biochar increased WR_0.25_ in which C_10_ significantly increased it (*p* < 0.05) by 15.85% and 14.28% for the N_100_ and N_80_ treatments, respectively, compared with C_0_ (Table 3).

As shown in Table 3, compared with no biochar, non-significant differences in the D-MWD and D-GMD were observed after the application of biochar for the each nitrogen fertilizer treatment (*p* > 0.05). However, biochar increased W-MWD and W-GMD for the each nitrogen fertilizer treatment, and compared with the C_0_, the W-MWD and W-GMD of C_10_ for the N_100_ treatment significantly increased by 17.08% and 42.22%, respectively (*p* < 0.05); and the W-MWD and W-GMD of C_10_ for the N_80_ treatment significantly increased by 14.88% and 36.26%, respectively (*p* < 0.05).

After the application of biochar, PAD_0.25_ for the each nitrogen fertilizer slightly decreased. The PAD_0.25_ of C_10_ for the each nitrogen fertilizer treatment was the lowest basically which decreased by 7.44%–34.74% as compared to C_0_, but there was no significant difference (*p* > 0.05) (Table 3). In general, biochar could enhance the water-stability of soil aggregates for the each nitrogen fertilizer, while the application of 10 t·ha^−1^ biochar served this purpose better. In addition, the combined application of C_10_ and N_100_ or N_80_ had a better improving effect on soil water-stable aggregates, compared with N_60_.

### 3.3. Effect of Biochar and Nitrogen Fertilizer on Total Organic Carbon in Soil Mechanical-Stable Aggregates

**Figure 1:**
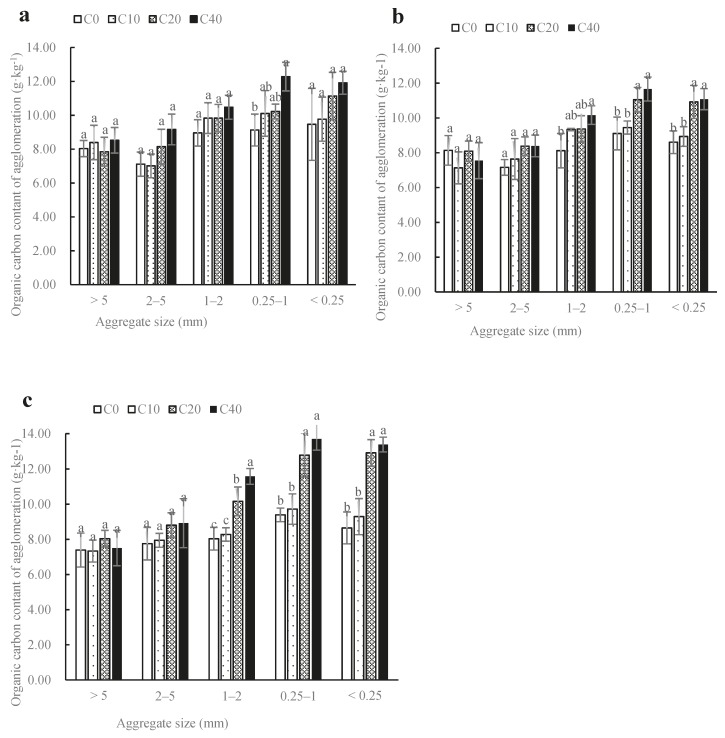
The content and distribution of organic carbon in mechanical-stable aggregates in the soil with biochar combined with conventional nitrogen application (**a**), 80% of conventional nitrogen application (**b**) and 60% of conventional nitrogen application (**c**). Different lowercase letters indicate significant differences among biochar treatments at the same nitrogen fertilizer at LSD_0.05._

As shown in Figure 1, TOC content in macro-aggregates (>0.25 mm) of soil mechanical-stable aggregates were basically all increased after biochar incorporation into soil for each nitrogen fertilizer treatment, and had an increasing trend with biochar increase. For N_100_ treatment, only the TOC content of C_40_ in the 0.25–1 mm aggregates was significant higher than that of C_0_ and the increases was 35.00% (*p* < 0.05). Nevertheless, for N_80_ and N_60_ treatments, C_20_ and C_40_ all caused a significant increase in TOC content in 0.25–1 mm and 1–2 mm aggregates (*p* < 0.05), compared with C_0_ (except for N_80_C_20_ treatment in the 1–2 mm aggregate). In the 0.25–1 mm aggregates, the TOC content were 11.05 g·kg^−1^ (N_80_C_20_), 11.66 g·kg^−1^ (N_80_C_40_), 12.78 g·kg^−1^ (N_60_C_20_) and 13.69 g·kg^−1^ (N_60_C_40_) after the application of biochar, and the increase rates were 21.00%, 28.00%, 36.00% and 46.00%, under N_80_C_20_, N_80_C_40_, N_60_C_20_ and N_60_C_40_, respectively, compared with the treatments of C_0_ combined with same nitrogen fertilizer application rate. Similarly, in the 1–2 mm aggregates, the biochar increased the TOC content by 15.00% (N_80_C_20_), 25.00% (N_80_C_40_), 27.00% (N_60_C_20_) and 44.00% (N_60_C_40_). In the <0.25 mm aggregates, the application of biochar also increased the TOC content. This indicated that the application of biochar for each nitrogen fertilizer treatment could increase the TOC content of soil mechanical-stable aggregates. In particular the increases of TOC content in the <2 mm aggregates were more remarkable and the effect of higher application of biochar (20 t·ha^−1^ or 40 t·ha^−1^) on increasing the TOC content was better.

### 3.4. Effect of Biochar and Nitrogen Fertilizer on Rapeseed Yield

**Figure 2:**
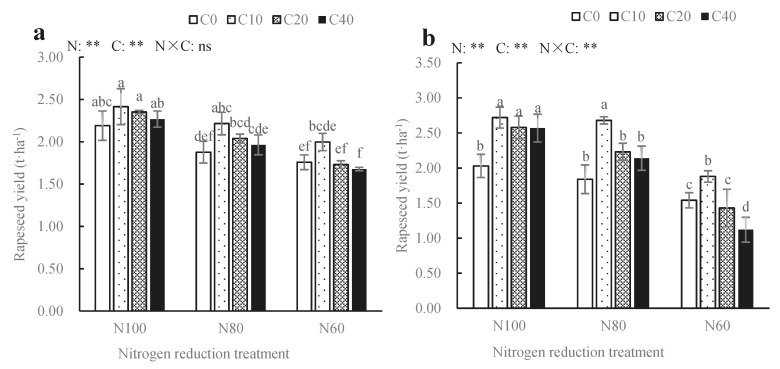
Rapeseed yield of biochar combined with reducing nitrogen application rate in 2018 (**a**) and 2019 (**b**). Different lowercase letters indicate significant differences at the different treatments at LSD_0.05_. N means nitrogen fertilizer; C means biochar; the “ns” means the difference is not significant and the “**” indicate significant difference at *p* < 0.01.

Figure 2 shows that biochar and nitrogen fertilizer significantly affected rapeseed yield in 2018 and 2019 (*p* < 0.01). Compared with no biochar, biochar increased rapeseed yield for N_100_ and N_80_ treatments, while only N_80_C_10_ treatment significantly increased (*p* < 0.05) rapeseed yield in the first year after biochar was applied (2018), being increased by 18.03% as compared to N_80_C_0_. Two years later (2019), compared with N_100_C_0_, N_100_C_10_, N_100_C_20_ and N_100_C_40_ significantly increased (*p* < 0.05) rapeseed yields by 33.99%, 27.09% and 26.60% respectively. For N_60_ and N_80_ treatment, only C_10_ significantly increased rapeseed yield (*p* < 0.05) by 22.08% and 45.65%, respectively, as compared to C_0_. However, rapeseed yield was slightly decreased after the application of high biochar rate (C_20_ and C_40_) in the N_60_ treatment, and C_40_ significant decreased (*p* < 0.05) rapeseed yield in 2019. These results showed that compared with N_60_, the combination of biochar with N_80_ and N_100_ was more conducive to the increase of rapeseed yield, especially at the optimal dosage of 10 t·ha^−1^.

For the same biochar application, the reduction of nitrogen fertilizer (N_80_ and N_60_) reduced the two-year rapeseed yield by 11.67% and 31.67%, respectively, compared with that of N_100_. Interactive effects of biochar and nitrogen fertilizer were also highly significant in 2019 (*p* < 0.01), and the highest yield of rapeseed was obtained by the combination of 10 t·ha^−1^ biochar and 180 kg·ha^−1^ pure nitrogen in two consecutive years, which had no statistical difference with N_80_C_10_ (*p* > 0.05). However, the two-year yields of the N_80_C_10_ treatment were all higher than those of N_100_C_0_, and the difference of yield between them reached a significant level in 2019 (*p* < 0.05). Compared with N_100_C_0_, the two-year average yield of N_80_C_10_ was increased by 16.11%. This indicated that the application of 10 t·ha^−1^ biochar could replace 20% pure nitrogen to still achieve high yield as compared to conventional nitrogen application.

### 3.5. Correlation of Soil Aggregate Stability with Rapeseed Yield

**Figure 3:**
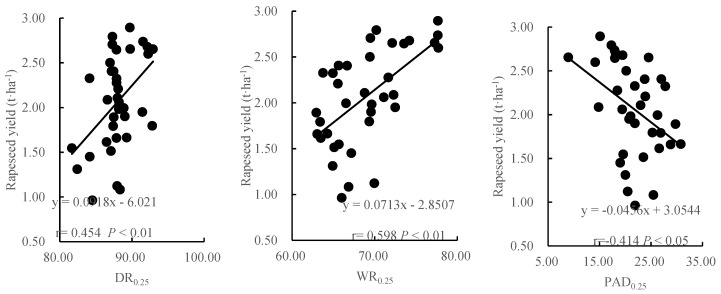

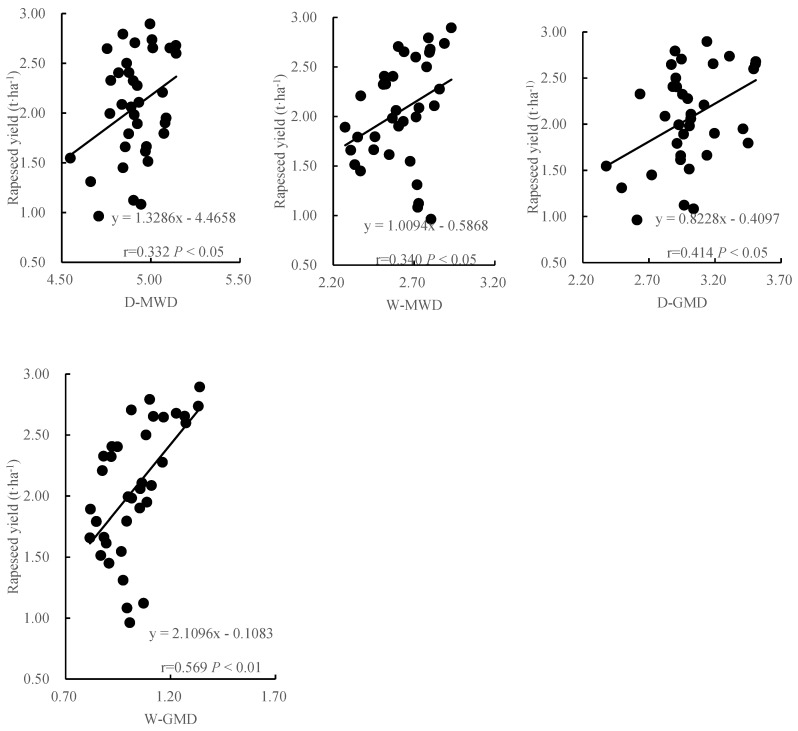
Relationship between soil aggregate index and rapeseed yield WR_0.25_ means content of >0.25 mm soil water-stable aggregates; DR_0.25_ means content of >0.25 mm soil mechanical-stable aggregates; W-MWD means mean weight diameter of water-stable aggregates; D-MWD means mean weight diameter of mechanical-stable aggregates; W-GMD means geometric mean diameter of water-stable aggregates; D-GMD means geometric mean diameter of mechanical-stable aggregates and PAD_0.25_ means >0.25 mm percentage of aggregate destruction.

In the present study, the regression analysis was performed to examine the relationships between indicators of soil structure stability (DR_0.25_, WR_0.25_, PAD_0.25_, D-MWD, D-GMD, W-MWD and W-GMD) and rapeseed yield in 2019 (Figure 3). DR_0.25_, WR_0.25_ and W-GMD had the strongest positive associations with rapeseed yield (*p* < 0.01), with the *r* values of 0.454, 0.598 and 0.569, respectively. D-MWD, D-GMD and W-MWD were also significantly related with rapeseed yield (*p* < 0.05), with correlation coefficients of 0.332, 0.340 and 0.414, respectively, whereas PAD_0.25_ had a significant negative correlation with rapeseed yield (*p* < 0.05; r = −0.414).

### 3.6. The Relationship between Rapeseed Yield and Application Rates of Biochar-Nitrogen Fertilizer

To study the relationship between rapeseed yield and application rates of biochar and nitrogen fertilizer, the multidimensional nonlinear regression model was built, in which biochar and nitrogen fertilizer were defined as independent variables and rapeseed yield was defined as dependent variable. The multidimensional nonlinear regression model was shown below:

*z* = −0.6741 + 30.3611*x* − 0.0027*y* + 0.1983*xy* − 81.3401*x*^2^ − 0.0007*y*^2^ (*R*^2^ = 0.831). (5)

**Figure 4:**
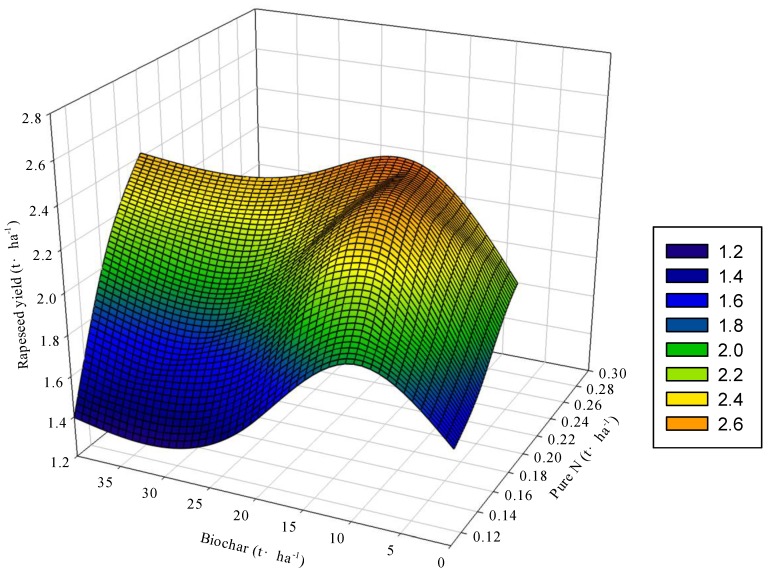
The relationship between rapeseed yield and application rates of biochar and N fertilizer in a 3D color map surface image.

In this equation, *x* is the nitrogen fertilizer application rate (t·ha^−1^); *y* is biochar application rate (t·ha^−1^); *xy* is the interactive effects of biochar and nitrogen fertilizer and *z* is the rapeseed yield (t·ha^−1^). Based on the equation, the 3D color map surface was built. As shown in Figure 4, the combined application of biochar and nitrogen fertilizer could increase rapeseed yield, while the increased rate of rapeseed yield tended to be gradual with biochar and nitrogen fertilizer increasing, and excessive application of biochar and nitrogen fertilizer even decreased the rapeseed yield. From the multidimensional regression model, it could be seen that the partial regression coefficient of nitrogen application rate (30.3611), which could reflect the contribution of nitrogen fertilizer to rapeseed yield, was greater than that of biochar application rate (0.0027). This indicated that the effect of biochar on rapeseed yield was smaller than that of nitrogen fertilizer. Moreover, the partial regression coefficient of nitrogen fertilizer and biochar combined with nitrogen fertilizer were greater than zero, indicating that nitrogen fertilizer and biochar combined with nitrogen fertilizer had positive effect on rapeseed yield. On the contrary, biochar had a slight inhibitory effect on rapeseed yield, indicating that a certain amount of nitrogen fertilizer could be combined to increase rapeseed yield. By computing the multi-dimensional regression models, the highest yield of rapeseed would be 2.67 t·ha^−1^, which could be achieved with a combined application of biochar and nitrogen fertilizer of 30 t·ha^−1^ and 223 kg·ha^−1^, respectively. By comparison with the highest yield, N_80_C_10_ (with yield of 2.45 t·ha^−1^) would reduce 35.43% nitrogen fertilizer in the case of small yield difference. It showed that the application of 10 t·ha^−1^ biochar could replace 35.43% pure nitrogen to achieve stable production compared the management combination with the highest yield.

## 4. Discussion

### 4.1. Effect of Biochar and Nitrogen Fertilizer on Content and Distribution of Soil Aggregates

Soil aggregates are the basic units of soil structure as well as the material basis of soil fertility, whose content and distribution are considered to be an important indicator of good soil aggregate structure, and play an important role in maintaining soil productivity. Organic fertilizer combined with inorganic fertilizer [19], straw returning or adding biochar [6] and conservation tillage [20] all increased the proportion of soil macroaggregates (>0.25 mm) and consequently increased soil aggregate stability. In this study, compared with no biochar, biochar increased content of soil mechanical-stable aggregates in 1–2 mm size and soil water-stable aggregates in >5 mm size for each nitrogen fertilizer. Besides, application of 10 t·ha^−1^ biochar overall significantly increased the soil water-stable aggregates in 2–5 mm size for each nitrogen fertilizer with an increase rate of 17.04%–33.04%, because of a corresponding decline in the percentage of micro-aggregates (<0.25 mm). This agreed with study results of Liu et al. [19], who found that the soil water stable aggregate (>0.25 mm) in the 0–15 cm soil layer had a remarkable increase after biochar application of 40 t·ha^−1^. This may be because biochar can adsorb and fix a variety of inorganic ions and polar or non-polar organic compounds, and then form organic–inorganic composites and large-size aggregates in soil due to its porous structure and large specific surface area [21]. In addition, biochar can produce organic matter such as polysaccharides and proteins by decomposition, and also produces more secretions due to increase of microbial activity in soil, thereby promotes the formation of soil large aggregates [22]. However, Dong et al. [6] found that the degree of soil aggregation in sandy loam had no significant change after three years of biochar application, possibly because of the difference in soil types. Compared with silty soil, sandy soil has poor adsorption capacity for soil organic carbon, and it is difficult to form a stable organic-inorganic composite with macromolecular organic matter [11], thus causing different response of soil aggregation to biochar.

However, in this study, higher biochar application (20 or 40 t·ha^−1^) was found to have no significant effect on the content of soil large aggregates. This might be because biochar could increase the soil total porosity and the soil water conservation capacity, and the soil total porosity was increased with the increase of biochar application rate [11]. The sampling time in this study was during the ripening stage of rapeseed, immediately after the end of the rainy season, and the soil in surface layer (0–20 cm) was greatly affected by rainfall, which increased the water content of surface soil, and thus decreased the soil cohesive force causing soil to be easily dispersed. In addition, biochar produced by high temperature with highly carboxylated and stable aromatization structure is mainly composed of inert carbon, which is difficult to be decomposed and utilized by microorganisms, and may not produce mucus aggregate particles [23]. Furthermore, biochar reduces the activity of enzymes related to organic carbon turnover due to adsorption of soil enzymes by biochar [24]. Therefore, under our experimental conditions, the effect of higher biochar application on formation of soil large aggregates was not significant. 

### 4.2. Effect of Biochar and Nitrogen Fertilizer on Soil Stability Index

R_0.25_, MWD and GMD are common indicators to reflect the size distribution of soil aggregates. Greater R_0.25_, MWD and GMD indicate higher stability of soil aggregate structure [25]. In this study, biochar amendment at 10 t·ha^−1^ significantly increased WR_0.25_, W-MWD and W-GMD under the treatments of conventional pure nitrogen application and 80% of conventional pure nitrogen application treatments as well as decreased the destruction of macroaggregates, which suggested that the positive effect of biochar combined with nitrogen fertilizer on increasing aggregate stability and improving soil structure in the purple soil. It can be seen from Table 1 that 10 t·ha^−1^ biochar significantly promoted the formation of soil water-stable aggregates in 2–5 mm size range under conventional nitrogen application and 80% of conventional nitrogen application, thereby significantly increased soil aggregate stability. However, Busscher et al. [26] found no significant change in aggregate content after 70 days of different amounts of biochar application. The main reason for these differences may be related to the differences in soil texture and soil organic matter content. In addition, the formation of soil aggregates requires an appropriate duration of time, and many studies are based on short-term tests, which may be another reason for the different test results. Therefore, the effect of biochar on the stability of soil aggregates requires more in-depth research.

However, 10 t·ha^−1^ biochar had no significant effect on soil WR_0.25_, W-MWD and W-GWD under the treatment of 60% of conventional nitrogen application, because there was no significant increase in content of soil water-stable aggregates in 2–5 mm size range after adding 10 t·ha^−1^ biochar, which might be related to the reduction in content of soil water-stable aggregates in 2–5 mm size range resulted from nitrogen reduction (Table 2). Li et al. [15] found that biochar and nitrogen fertilizer had significant interaction effects on the stability of soil aggregates. Compared with conventional fertilization, no biochar and the 1 t·ha^−1^ biochar application under the treatment of nitrogen fertilizer reduction significantly reduced water-stable aggregates, but there was no significant difference between the 5 and 10 t·ha^−1^ biochar application. This suggests that the application of higher biochar may be more conducive to the stability of water-stable aggregates when soil fertility is low or conventional nitrogen fertilizer is reduced.

### 4.3. Effect of Biochar and Nitrogen Fertilizer on Total Organic Carbon in Soil Mechanical-Stable Aggregates

SOM, as an important aggregate binding agent, plays an important role in the formation of large aggregates and the improvement of soil structure [27]. This study demonstrated that biochar amendment led to an increase in soil organic carbon in 2–5, 1–2, 0.25–1 and <0.25 mm aggregates for each nitrogen fertilizer treatment, and SOC in 2–5, 1–2, 0.25–1 and <0.25 mm aggregates increased with the increase in biochar application. This was because the organic carbon content of biochar used in this experiment was 537.97 g·kg^−1^, being a carbon-rich exogenous organic matter, thus it could increase the soil microbial biomass and promote the formation of soil humus to contribute to the formation of organic macromolecules such as carbohydrates and aromatic hydrocarbons, thereby increasing the content of soil organic carbon and producing more cementing materials, finally resulting in the redistribution of microaggregates in the soil into large aggregates [28]. This was also the reason why the application of 10 t·ha^−1^ biochar in this study promoted the formation of soil large aggregates and thus improved the stability of soil aggregates.

By analyzing the organic carbon content of aggregates in each size range, we could clearly understand the outward distribution of exogenous new carbon in soil aggregates and its influence on soil aggregate stability, which may help to reveal the sequestration and protection mechanism of soil organic carbon. Gao et al. [29] showed that the organic carbon content of soil aggregates increased as the aggregate fraction size increased, and large aggregates could absorb more carbon. However, in our study, soil organic carbon was mainly stored in soil aggregates of 0.25–1 and <0.25 mm size ranges, and high level of biochar (20 or 40 t·ha^−1^) significantly increased the total organic carbon of these two fractions for each nitrogen treatment. This might be because in this experiment, conventional tillage was frequently carried out by mechanical tillage and caused breakage of soil large aggregates (>5 and 2–5 mm), thus they became easily attacked by microorganism, resulting in accelerating mineralization of SOM, and the retention of SOM was unfavorable [30]. However, through the action of stabilizing force, biochar combined with soil minerals to form aggregates, whose physical protection could reduce the decomposition of soil organic carbon in aggregates of 1–2, 0.25–1 and <0.25 mm size ranges by microorganism. At the same time, colloidal components with high degree of humification such as humic acid and fulvic acid became relatively enriched in micro-aggregates, and new carbon with high degree of humification or aromatic carbon, which was difficult to be utilized by microorganisms was stably stored by wrapping effect of powdery minerals [31].

### 4.4. Effect of Biochar and Nitrogen Fertilizer on Rapeseed Yield

Soil aggregate structure is not only an important factor in plant growth and water transport in soil, but also plays an important role in soil physical and chemical properties and biological processes [32]. Early studies have shown that more stable soil aggregate structure indicates stronger soil ability to resist erosion, and higher soil fertility and crop yield [27]. Our experiment could also prove this result since the stability of soil aggregates was significantly correlated with rapeseed yield (Figure 3). The larger the values of R_0.25_, MWD and GMD were, the smaller the aggregate destruction rate was and the higher the rapeseed yield was. In our experiment, biochar with application rate of 10 t·ha^−1^ for 2 years under different nitrogen fertilizer treatments significantly increased rapeseed yields by 22.08%–45.65% as compared to no biochar treatments. This was because some indicators as evaluating soil quality (R_0.25_, MWD, GMD and PAD_0.25_) were improved after the biochar application, which reflected that biochar combined with nitrogen fertilizer significantly improved the stability of soil aggregates. This indicated that stable soil aggregates could provide a good soil environment for crop growth, thus increasing the nutrient utilization and yield of crops. Yuan et al. [16] also showed that the combined application of biochar and nitrogen fertilizer improved the stability of soil aggregates, and the physical and chemical properties of soil and micro-ecological environment also had obvious improvement, which provided favorable growth conditions for jujube, and thus increasing the jujube yield.

This study also found that the interaction effect of biochar and nitrogen fertilizer on rapeseed yield was positively significant (Figure 2 and Figure 4). In all treatments, rapeseed yield of the N_100_C_10_ treatment was the highest, which had no significant difference with that of the N_80_C_10_ treatment in 2 years. However, rapeseed yield of N_80_C_10_ treatment was significantly higher than that of conventional fertilization treatment, with the average yield in 2 years increased by 16.11%. This indicated that compared with the conventional fertilization, 10 t·ha^−1^ biochar could replace 20% of nitrogen fertilizer to achieve high yield, which have been proven by many researchers [33]. At the same time, the combined application of 30 t·ha^−1^ biochar and 223 kg·ha^−1^ nitrogen fertilizer could achieve the highest rapeseed yield (2.67 t·ha^−1^) by the analysis of multivariate nonlinear regression equations, which was only 8.98% higher than rapeseed yield of the N_80_C_10_ treatment, but the N_80_C_10_ treatment could reduce 35.43% nitrogen fertilizer. Based on the results of this experiment, 10 t·ha^−1^ biochar combined with 144 kg·ha^−1^ pure nitrogen (80% of conventional nitrogen) is conducive not only to enhance the stability of soil aggregates in purple soil and increase the fixation of organic carbon, but also to improve rapeseed yield greatly under the condition of 20% reduction of nitrogen fertilizer. This study will not only improve the capacity of fixed carbon and reducing discharge in farmland and achieve the coordination of agricultural production and ecological functions, but also greatly decrease water and soil pollution from nitrogen fertilize.

This study mainly focused on the study of the physical and chemical properties of soil aggregates under different application rates of biochar and nitrogen fertilizer. It is necessary to further study soil biological properties, so as to better reveal the variation mechanism of aggregate composition and organic carbon distribution in purple soil, which could provide a scientific basis for the rational application of biochar in agricultural production.

## 5. Conclusions

Biochar (10 t·ha^−1^) significantly increased soil water-stable aggregates of 2–5 mm size, WR_0.25_, W-MWD and W-GMD for conventional nitrogen application and 80% of conventional nitrogen application treatments, but reduced the breakage of soil macro-aggregates and consequently increased soil aggregate stability. Moreover, biochar also significantly increased the TOC in soil mechanical-stable aggregates under each nitrogen fertilizer treatment, especially the TOC in soil aggregates of 1–2, 0.25–1 and <0.25 mm size ranges. Reducing nitrogen application rate decreased rapeseed yield, while applying 10 t·ha^−1^ biochar into soil, rapeseed yield for each nitrogen fertilizer treatment was significantly increased and 20% of nitrogen fertilizer could be replaced when compared with conventional nitrogen application. Compared with the combination of biochar and nitrogen fertilizer under which the highest yield (2.67 t·ha^−1^) could be achieved according to the analysis of multidimensional regression model, the application of 10 t·ha^−1^ biochar combined with 80% of conventional nitrogen fertilizer could save 35.43% of nitrogen fertilizer input, while the yield only decreased by 8.24%. The present study suggested a great potential of applying 10 t·ha^−1^ biochar combined with 144 kg·ha^−1^ pure nitrogen to achieve favorable soil structure with sustaining rapeseed productivity in upland purple soils in southwest China.

## Figures and Tables

**Table 1:** Results of two-factor ANOVA (F values) with biochar and nitrogen fertilizer as independent factors for changes in soil attributes.

Factors	Soil Aggregates by Dry Sieving					Soil Aggregates by Wet Sieving					DR_0.25_	D-MWD	D-GMD	WR_0.25_	W-MWD	W-GMD	PAD_0.25_
	>5	2–5	1–2	0.25–1	<0.25	>5	2–5	1–2	0.25–1	<0.25							
N	ns	ns	ns	*	ns	ns	**	ns	ns	*	ns	ns	ns	*	ns	ns	ns
C	**	ns	**	ns	**	**	**	**	ns	**	**	**	**	**	**	**	ns
N × C	ns	ns	ns	ns	ns	ns	ns	ns	ns	ns	ns	ns	ns	ns	ns	ns	ns

Note: The “ns” means the difference is not significant; the “*” and “**” indicate significant difference at *p* < 0.05 and *p* < 0.01, respectively. N means nitrogen fertilizer; C means biochar; WR_0.25_ means content of >0.25 mm soil water-stable aggregates; DR_0.25_ means content of >0.25 mm soil mechanical-stable aggregates; W-MWD means mean weight diameter of water-stable aggregates; D-MWD means mean weight diameter of mechanical-stable aggregates; W-GMD means geometric mean diameter of water-stable aggregates; D-GMD means geometric mean diameter of mechanical-stable aggregates; PAD_0.25_ means >0.25 mm percentage of aggregate destruction.

**Table 2:** The effect of biochar and nitrogen fertilizer on content and distribution of soil aggregates.

Treatments		Aggregate Size (mm), %									
Pure N (kg·ha^−1^)	Biochar (t·ha^−1^)	Dry Sieving					Wet Sieving				
		>5	2–5	1–2	0.25–1	<0.25	>5	2–5	1–2	0.25–1	<0.25
180 (N_100_)	0 (C_0_)	54.0 ± 1.8Aa	22.5 ± 1.1Aa	5.0 ± 0.4Aa	6.5 ± 0.3Aa	12.1 ± 0.4Aab	17.6 ± 1.3Aa	23.0 ± 2.0Ab	8.7 ± 0.7Aa	16.7 ± 1.2Aa	34.0 ± 3.4Aa
	10 (C_10_)	55.1 ± 0.4Aa	23.0 ± 2.3Aa	6.6 ± 0.6Aa	6.6 ± 0.5Aa	8.6 ± 1.4Aa	19.6 ± 2.4Aa	30.6 ± 1.7Aa	9.1 ± 0.9Aa	17.2 ± 1.5Aa	23.5 ± 2.0Ab
	20 (C_20_)	51.2 ± 1.8Aa	24.4 ± 1.4Aa	5.3 ± 0.4Aa	6.7 ± 0.5Aa	12.5 ± 0.3Aa	21.0 ± 0.9Aa	23.4 ± 1.4Ab	7.8 ± 1.4Aa	17.7 ± 0.5Aa	30.1 ± 3.5Aab
	40 (C_40_)	52.4 ± 0.9Aa	22.8 ± 1.7Aa	5.1 ± 0.4Aa	6.9 ± 0.5Aa	12.8 ± 0.2Aa	21.5 ± 1.6Aa	23.7 ± 1.1Ab	7.5 ± 0.2Aa	15.6 ± 0.5Aa	31.6 ± 2.4Aa
144 (N_80_)	0 (C_0_)	52.0 ± 0.4Aa	24.0 ± 1.0Aa	5.0 ± 0.7Aa	6.8 ± 0.7Aa	12.2 ± 0.4Aa	17.9 ± 0.9Ab	22.7 ± 2.3Ab	9.0 ± 1.0Aa	16.3 ± 1.2Aa	34.2 ± 4.6Aa
	10 (C_10_)	54.1 ± 0.6Aa	23.8 ± 0.4Aa	6.6 ± 1.0Aa	7.0 ± 0.1Aa	8.6 ± 1.6Aa	19.6 ± 1.7Aab	29.4 ± 1.6Aa	9.2 ± 1.2Aa	17.5 ± 1.3Aa	24.3 ± 3.1Ab
	20 (C_20_)	53.0 ± 0.4Aa	22.0 ± 0.9Aa	5.4 ± 0.6Aa	7.0 ± 0.3Aa	12.5 ± 0.7Aa	21.4 ± 1.7Aab	23.7 ± 1.9Ab	8.5 ± 1.0Aa	16.0 ± 2.2Aa	30.4 ± 4.1Aab
	40 (C_40_)	51.8 ± 1.7Aa	22.9 ± 2.2Aa	5.2 ± 0.9Aa	7.1 ± 0.9Aa	13.0 ± 2.5Aa	22.8 ± 1.6Aa	20.9 ± 2.2Ab	7.2 ± 1.2Aa	15.5 ± 0.7Aa	33.6 ± 2.5Aa
108 (N_60_)	0 (C_0_)	53.6 ± 0.7Aa	22.5 ± 1.2Aa	5.0 ± 0.6Ab	5.8 ± 0.6Aa	13.2 ± 2.6Aab	17.6 ± 1.3Ac	22.3 ± 0.8Aab	9.5 ± 0.9Aa	16.2 ± 1.2Aa	34.5 ± 1.5Aa
	10 (C_10_)	55.0 ± 1.0Aa	22.7 ± 1.9Aa	7.3 ± 0.7Aa	6.1 ± 0.9Aa	8.9 ± 1.9Ab	18.2 ± 1.5Abc	26.1 ± 1.2Ba	9.8 ± 0.8Aa	16.4 ± 2.4Aa	29.5 ± 1.8Aab
	20 (C_20_)	52.2 ± 3.3Aa	21.6 ± 1.2Aa	5.3 ± 0.5Ab	6.3 ± 0.9Aa	14.6 ± 3.3Aa	21.9 ± 1.0Aab	21.4 ± 1.7Ab	8.2 ± 0.7Aa	14.8 ± 1.0Aa	33.6 ± 3.3Aa
	40 (C_40_)	51.6 ± 1.6Aa	21.9 ± 0.9Aa	5.2 ± 0.3Ab	6.4 ± 0.8Aa	14.9 ± 3.0Aa	23.9 ± 1.1Aa	20.5 ± 1.3Ab	7.1 ± 0.3Aa	14.4 ± 1.4Aa	34.1 ± 1.0Aa

Note: the letters A, B and a, b and c in a single column indicate significant difference under different nitrogen and biochar levels, respectively, using LSD test at *p* < 0.05.

**Table 3:** Changes in soil aggregate (>0.25 mm) content, mean weight diameter, geometric mean diameter and percentage of aggregate destruction with different treatments.

Treatments		DR_0.25_	D-MWD	D-GMD	WR_0.25_	W-MWD	W-GMD	PAD_0.25_
Pure N (kg·ha^−1^)	Biochar (t·ha^−1^)							
180	0	87.93 ± 0.38Aa	4.97 ± 0.09Aa	3.03 ± 0.08Aa	66.05 ± 3.38Ab	2.40 ± 0.15Ab	0.90 ± 0.10Ab	24.90 ± 4.46Aa
	10	91.38 ± 1.43Aa	5.09 ± 0.08Aa	3.38 ± 0.21Aa	76.52 ± 2.02Aa	2.81 ± 0.11Aa	1.28 ± 0.06Aa	16.25 ± 2.85Aa
	20	87.55 ± 0.30Aa	4.83 ± 0.08Aa	2.91 ± 0.04Aa	69.90 ± 3.46Aab	2.66 ± 0.13Aab	1.04 ± 0.11Ab	19.96 ± 3.31Aa
	40	87.16 ± 0.17Aa	4.86 ± 0.02Aa	2.89 ± 0.01Aa	68.42 ± 2.45Ab	2.69 ± 0.15Aab	1.04 ± 0.10Ab	21.50 ± 4.99Aa
144	0	87.83 ± 0.41Aa	4.87 ± 0.02Aa	2.96 ± 0.06Aa	65.85 ± 4.56Ab	2.42 ± 0.15Ab	0.91 ± 0.13Ab	25.04 ± 4.97Aa
	10	91.42 ± 1.59Aa	5.04 ± 0.06Aa	3.33 ± 0.16Aa	75.68 ± 3.08Aa	2.78 ± 0.13Aa	1.24 ± 0.11ABa	17.13 ± 7.84Aa
	20	87.46 ± 0.73Aa	4.89 ± 0.04Aa	2.92 ± 0.09Aa	69.63 ± 4.07Aab	2.70 ± 0.18Aab	1.06 ± 0.13Aab	20.35 ± 6.69Aa
	40	87.01 ± 2.52Aa	4.83 ± 0.09Aa	2.86 ± 0.20Aa	66.36 ± 2.52Ab	2.69 ± 0.15Aab	0.98 ± 0.09Ab	23.70 ± 2.29Aa
108	0	86.85 ± 2.56Aab	4.93 ± 0.08Aab	2.95 ± 0.21Aab	65.52 ± 1.49Aa	2.39 ± 0.06Ab	0.89 ± 0.02Aa	24.45 ± 5.95Aa
	10	91.11 ± 1.92Aa	5.08 ± 0.01Aa	3.35 ± 0.13Aa	70.47 ± 1.75Aa	2.57 ± 0.09Aab	1.04 ± 0.05Ba	22.63 ± 2.37Aa
	20	85.38 ± 3.28Ab	4.81 ± 0.23Aab	2.76 ± 0.33Ab	66.37 ± 3.29Aa	2.65 ± 0.09Aab	0.98 ± 0.09Aa	22.22 ± 3.82Aa
	40	85.11 ± 3.01Ab	4.77 ± 0.16Ab	2.71 ± 0.29Ab	65.93 ± 0.97Aa	2.75 ± 0.05Aa	0.99 ± 0.02Aa	22.47 ± 2.77Aa

Note: the letters A, B and a and b in a single column indicate significant difference under different nitrogen and biochar levels, respectively, using LSD test at *p* < 0.05. WR_0.25_ means content of >0.25 mm soil water-stable aggregates; DR_0.25_ means content of >0.25 mm soil mechanical-stable aggregates; W-MWD means mean weight diameter of water-stable aggregates; D-MWD means mean weight diameter of mechanical-stable aggregates; W-GMD means geometric mean diameter of water-stable aggregates; D-GMD means geometric mean diameter of mechanical-stable aggregates; PAD_0.25_ means >0.25 mm percentage of aggregate destruction.
